# Analysis of reactive astrogliosis in mouse brain using *in situ* hybridization combined with immunohistochemistry

**DOI:** 10.1016/j.xpro.2021.100375

**Published:** 2021-03-02

**Authors:** Ranjithmenon Muraleedharan, Diana Nardini, Ronald Raymond Waclaw, Biplab Dasgupta

**Affiliations:** 1 Division of Oncology, Cincinnati Children’s Hospital Medical Center, Cincinnati, OH 45229, USA; 2 Division of Experimental Hematology and Cancer Biology, Cincinnati Children’s Hospital Medical Center, Cincinnati, OH 45229, USA; 3 Department of Pediatrics, University of Cincinnati College of Medicine, Cincinnati, OH 45229, USA

**Keywords:** Cancer, Metabolism, Microscopy, Model organisms, *In situ* hybridization, Neuroscience

## Abstract

Reactive astrogliosis is characterized by a profound change in astrocyte phenotype in response to all CNS injuries. Here, we present a revised *in situ* hybridization and immunohistochemistry (IHC) protocol to label the reactive astrocytes in the mouse brain. Several approaches for quantifying astrocyte reactivity lacked sensitivity to discriminate across the spectrum. We optimized *in situ* hybridization followed by IHC. We provide a staining protocol for quantitative measures of astrocyte reactivity as an independent confirmation of the magnitude of reactive gliosis.

For complete details on the use and execution of this protocol, please refer to Muraleedharan et al. (2020).

## Highlights

• Quantitative measurement of reactive gliosis in mouse brain
• Dual staining identifies reactive gliosis occurs in response to chronic CNS pathology
• Hybridization phase includes LCN2 and C3 mRNA hybridization and signal development
• Immunostaining phase includes antibody detection of GFAP protein

## Graphical Abstract

**Figure:**
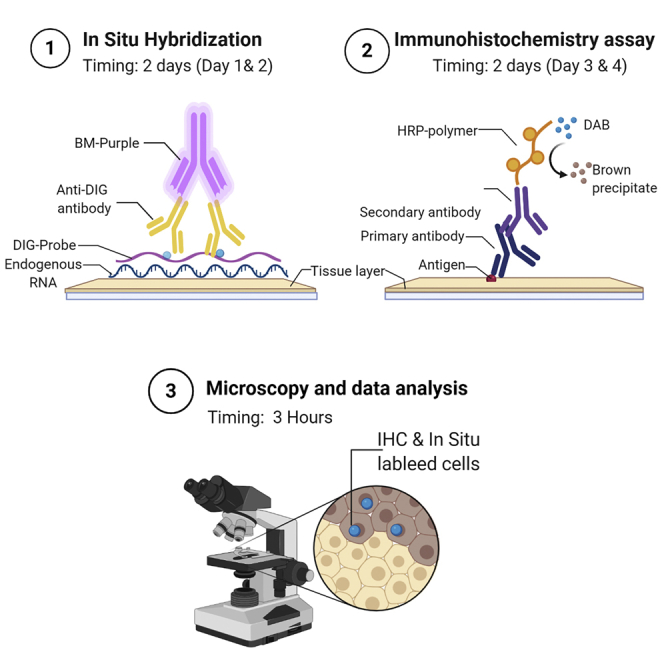

## Before you begin

This dual staining protocol was created to detect reactive astrocytes in mouse brain sections, allowing the distinction between normal and activated astrocytes. The LCN2 & C3 genes were chosen because they are widely validated genes used as an indirect marker of astrocytes activation in the brain. Here we employ newly designed probes to simultaneously detect LCN2 or C3 mRNA followed by immunostaining with GFAP antibody of high specificity for astrocytes.

The protocol detailed below describes the procedure for brain dissection and sectioning, *in situ* hybridization to detect LCN2 or C3, and immunostaining to detect GFAP protein. The comments outlining critical steps are provided.

***Note:*** The *in situ* hybridization protocol is a modification from the protocol published by Toresson et al., 1999 (Toresson et al., 1999) and Kohli et al., 2018 (Kohli et al., 2018).

### Designing the RNA probes

Optimal probe lengths are usually considered to be 300–500 bases, however, longer probes may result in slightly higher background labeling or may not penetrate tissue. RNA probes are PCR amplified from cDNA and generated with standard methods from Promega and Roche.

**CRITICAL:** All solutions, materials, and equipment should be RNase-free.

**CRITICAL:** Make sure you optimized the antibodies before you start the combined *in situ* hybridization and IHC protocol.

### RNA probe synthesis

**Timing: 1–2 days**

The following protocol describes the production of DIG labeled RNA probes.

**CRITICAL:** Throughout the protocol, work on a clean bench, use RNase/DNase-free tips, reagents, and tubes to avoid probe degradation.

1. PCR amplification of probe region from cDNA obtained from postnatal mouse brain RNA-PCR reaction with primers (Note: 3′ primer should contain the T3 sequence at the 5′ end)- Example for LCN2:5′ Primer – GCTGTCGCTACTGGATCAGA3′ Primer (T3 sequence in italics/bold) – ***ATTAACCCTCACTAAAGG*** TGGTGGTGTTAAGACAGGTGG

***Note:*** GoTaq from Promega is used for PCR amplification of region.

PCR reaction mix per sample. Run eight samples for one probe.

**Table:** 

GoTaq master mix	12.5 μL
Primer 1 (25 μM)	1 μL
Primer 2 (25 μM)	1 μL
cDNA (approx. 2,000 ng/μL)	0.75 μL
RNase-free water	9.75 μL

**Table:** 

PCR cycling conditions			
Steps	Temperature	Time	Cycles
Initial denaturation	98°C	30 s	1
Denaturation	98°C	20 s	35 cycles
Annealing	55°C	20 s	
Extension	72°C	1 min	
Final extension	72°C	10 min	1
Hold	4°C	Forever

2. Isolate and extract PCR product from 2% agarose gel using Qiagen Gel Extraction kit methods. https://www.qiagen.com/us/shop/pcr/qiaquick-gel-extraction-kit/
3. Prepare *in vitro* transcription reaction.

**Table:** 

PCR product of target region	1 μg template DNA
5× reaction buffer	8 μL
DIG labeling Mix dNTPs	4 μL
RNase inhibitor	1 μL
T3 RNA polymerase	3 μL
RNase-free water	fill up to 40 μL total volume

Incubate at 37°C for 4 h

4. Add 10× DNase Buffer (4 μL) and DNase I (1.5 μL) and incubate for 1 h at 37°C.
5. Stop probe reactions using Ammonium acetate/EDTA stop solution (15 μL; see the recipe for Stock Stop Solution in “Materials and equipment”), RNase-free water (95 μL), and cold Isopropanol (151 μL).
6. Place probe tubes at −20°C for 12–18 h.
7. Spin probe tubes at 21,130 × *g* for 20 min at 4°C, carefully remove supernatant and add 300 μL of 100% ethanol and spin again at 21,130 × *g* for 20 min at 4°C.
8. Carefully remove supernatant and dry pellet at 20°C–22°C for 5–7 min.
9. Resuspend pellet with 17 μL of RNase-free water.
10. Run 2 μL on a 1% agarose gel to check probe.
11. Store probe tubes at −80°C for long term storage (up to 6 months) or −20°C for short term storage (up to 72 h).

## Key resources table

**Table:** 

REAGENT or RESOURCE	SOURCE	IDENTIFIER
**Antibodies**		

Anti-GFAP antibody	DAKO	DAKO: Z0334
Anti-rabbit biotinylated secondary antibody	DAKO	DAKO- E0353
Anti-DIG antibody	Roche	11093274910

**Chemicals, peptides, and recombinant proteins**		

16% PFA	Electron Microscopy Sciences	15710
10× dPBS	Life Technologies	14200166
Proteinase K	Life Technologies	25530049
RNaseA	Invitrogen	12091021
Torula yeast RNA (tRNA)	Sigma-Aldrich	R6625-25G
Levamisol	Sigma-Aldrich	L9756-5G
BM-Purple	Roche	11442074001
Normal goat serum (NGS)	Jackson ImmunoResearch	005-000-121
20× SSC	Invitrogen	15557044
Dextran sulfate	Fisher Bioreagents	BP1585-100
50× Denhardt’s solution	Sigma-Aldrich	D2532-5mL
Formamide (high stringency)	Sigma-Aldrich	F7503-1L
Tween 20	Fisher Bioreagents	BP337-100
Formamide (Hyb buffer)	Sigma-Aldrich	F9037-100mL
NaCl	Fisher Bioreagents	BP358212
Tris-HCl	Fisher Bioreagents	BP1531
Tris base	Fisher Chemical	T3951
NaH_2_PO_4_ anhydrous	Sigma-Aldrich	S3139
Na_2_HPO_4_ anhydrous	Sigma-Aldrich	S3264
1 M Tris-HCl pH 7.5	Invitrogen	15567027
MgCl_2_	Sigma-Aldrich	M8266-1kG
Isopropanol	Fisher Chemical	A416500
Ammonium acetate	Sigma-Aldrich	A1542
Ultra Pure 0.5 M EDTA	Life Technologies	15575020
1 M Tris pH 9.5	Teknova	T1095
Sucrose solution	Sigma-Aldrich	S0389
Acetone	Fisher Chemical	A181
Hydrogen peroxide solution	Sigma	H1009-5ML
Triton X-100	Sigma	T8787

**Critical commercial assays**		

VECTASTAIN ABC Reagent	Vectorlabs	PK-6100
VECTASHIELD Vibrance Antifade Mounting Medium	Vectorlabs	H-1700-2
DAB substrate	Sigma-Aldrich	D5637
T3 RNA polymerase	Promega	P2083
5× reaction buffer	Promega	P1181
10× DNase buffer	Thermo Fisher formerly Ambion	AM8160G
DNase I	Thermo Fisher formerly Ambion	AM2235
RNase inhibitor	NEB	M0307S
GoTaq Master Mixes	Promega	M7122
Qiaquick Kit	Qiagen	28706
DIG DNA Labeling Mix (DIG labeling Mix dNTPs)	Millipore Sigma	11277065910

**Experimental models: organisms/strains**		

Mouse: male 4–24 months AMPKb1lox/lox AMPKb2-/- Nestin Cre	DasGupta Lab	N/A

**Oligonucleotides**		

LCN2-5′	IDT	GCTGTCGCTACTGGATCAGA
LCN2-3- with T3	IDT	ATTAACCCTCACTAAAGG TGGTGGTGT TAAGACAGGTGG
CompC3-5′	IDT	TGGGCAAGACAGTCGTCATC
CompC3-3′ with T3	IDT	ATTAACCCTCACTAAAGG TGGATCTGGT ACGGGGAAGT

**Software and algorithms**		

GraphPad Prism 8	GraphPad	RRID: SCR_002798 https://www.graphpad.com/
ImageJ	https://imagej.nih.gov/ij/download.html	ImageJ; RRID: SCR_003070
Leica Acquisition Software (LASX) suite	Leica	n/a

**Other**		

Richard-Allen Scientific Neg-50 Frozen Section Medium	Thermo Scientific	6502
Dry ice	n/a	n/a
Leica light microscope DM2500	Leica	n/a
Lecia DMC6200 camera	Leica	n/a
Leica cryostat	Leica	n/a
Superfrost slides	VWR	48311-703
Plastic molds disposable deep base molds, 37 × 24 × 10 mm, 500 PCS/pack	Ihcworld	M475-10

## Materials and equipment

### RNase-free pre-hybridization and hybridization solution

Store at −20°C for 6 months

***Note:*** 50% dextran sulfate solution is viscous and should be pipetted with care to ensure precision in volume measurements.

**Table:** 

Reagent	Final concentration	Amount
Deionized formamide	50% (v/v)	25 mL
50 mg/mL torula yeast tRNA	200 μg/mL	200 μL
50× Denhardt's solution	1×	1 mL
50% Dextran sulfate	10%	10 mL
Salt solution	10% (v/v)	5 mL
RNase-free ultrapure water	n/a	9 mL
Total		50 mL

### Salt solution

**Table:** Make 5 mL aliquots and store at −20°C for 6 months

Reagent	Final concentration	Amount
NaCl	3 M	17.5 g
Tris-Cl pH 8.0	0.089 M	1.4 g
Tris Base	0.011 M	0.14 g
NaH_2_PO_4_ anhydrous	0.05 M	0.6 g
Na_2_HPO_4_	0.05 M	0.71 g
EDTA pH 8.0 (0.5 M)	0.05 M	10 mL
RNase-free ultrapure water	n/a	Fill up to 100 mL

### Stock stop solution

**Table:** Stop solution can be aliquoted and stored at −20°C for 6 months.

Reagent	Final concentration	Amount
Ammonium acetate	5 M	1.927 g
EDTA (0.5 M)	0.1 M	1 mL
RNase-free ultrapure water	n/a	Fill up to 5 mL

### High-stringency wash buffer

**Table:** Stop solution can be aliquoted and stored at 22°C for 6 months.

Reagent	Final concentration	Amount
Formamide	50% (v/v)	125 mL
20× SSC	2×	25 mL
RNase-free ultrapure water	n/a	Fill up to 250 mL

### RNase buffer

**Table:** Stop solution can be aliquoted and stored at 22°C for 6 months.

Reagent	Final concentration	Amount
NaCl (5 M)	0.5 M	50 mL
Tris-HCl pH 7.5 (1 M)	0.01 M	5 mL
EDTA pH8 (0.5 M)	0.005 M	5 mL
RNase-free ultrapure water	n/a	Fill up to 500 mL

### NTMT buffer

**Table:** Stop solution can be aliquoted and stored at −20°C for 6 months.

Reagent	Final Concentration	Amount
NaCl (5 M)	0.1 M	3 mL
Tris-HCl pH 9.5 (1 M)	0.1 M	15 mL
MgCl_2_ (1 M)	0.05 M	7.5 mL
Tween 20	0.1%	150 μL
RNase-free ultrapure water	n/a	Fill up to 150 mL

## Step-by-step method details

### Collection and preparation of mouse brain for ISH-tissue dissection, fixing, and cryoprotection

**Timing: 2 days**

This step describes the protocol from dissecting mouse brain until it is fixed and ready for embedding. Keep the time between perfusion or sacrificing of the animal and fixation as short as possible, for best preservation of morphology.

1. Day 1: Animal procedure. Place mouse in isoflurane chamber to anesthetize the animal.
2. Prior to surgery, a ketamine/xylazine mixture dissolved in saline (up to 80 mg/kg body weight ketamine and 10 mg/kg body weight xylazine) is administered via intraperitoneal injection (27-gauge needle and 1 mL syringe).
3. Use toe pinch response method to determine depth of anesthesia. Animal must be unresponsive before continuing.
4. Once the animal has reached a surgical plane of anesthesia, place mouse on its back on the shallow tray.
5. Make a 5–6-cm lateral incision through the integument and abdominal wall just beneath the rib cage.
6. Continue the diaphragm incision along the entire length of the rib cage to expose the pleural cavity.
7. Insert the 30-gauge needle into the posterior end of the left ventricle, being careful to keep the tip of the needle in the lumen of the ventricle.
8. Finally, make an incision to the animal's right atrium using iris scissors to create as large an outlet as possible without damaging the descending aorta. At this point the animal is ready to be perfused.
9. Perfusion. Open and attach outlet port to needle base and pump up the manometer bulb to a pressure of 80 mm Hg.
10. Perfuse with saline solution for 10 min.
11. After 10 min, switch the stopcock to allow for flow of formalin solution, at same flow rate as saline.
12. Perfuse with 4% PFA solution for 10–15 min.
13. The mouse should be stiff at this stage.
14. Separate the mouse head with surgical scissors by making a cut posterior from the ears. Make a caudal midline incision in the skin and work rostrally to remove the skin from skull.
15. Starting from the caudal part, cut through the top of the skull along midline and between the eyes with Iris scissors. Remove the parietal and frontal bone plates by tilting one side of a bone plate each time and snapping it off with tweezers.
16. Gently tilt the brain upward from the anterior part with tweezers and cut the optic nerves and other cranial nerves. Gently lift the brain out of the skull.
17. Remove the brain and place it in a vial of fixative containing fluid at least 10× the volume of the brain itself. Swirl the vial occasionally.
18. Fix biopsies (3×3 mm) immediately in freshly prepared 4% (w/v) PFA/PBS for 12–18 h.

**CRITICAL:** When handling paraformaldehyde (PFA), both solid and aqueous, wear PPE and use a safety cabinet. Prepare formaldehyde solution by dissolving 4% (w/v) PFA into 1× phosphate buffered saline (PBS) solution using heat (55°C). Filter the formaldehyde solution with filter paper.

**CRITICAL:** Proper fixation protocol followed by an appropriate tissue preparation form the basis for good histology (Montero, 2003). The ideal fixative should preserve the antigens in the tissue in a form that is accessible and recognizable to the antibodies applied for the labeling technique.

19. Day 2: Cryoprotection. Following fixation, keep the brain in 15% sucrose for 2–3 h and 30% sucrose for 12–18 h.
20. Brain can be embedded for cryosectioning.

**CRITICAL:** This step is important for keeping the intact structure of the brain anatomy, including astrocytes morphology.

### Preparation of frozen tissue blocks/embedding and sectioning

**Timing: 2 days**

This part describes the preparation of frozen tissue blocks for the mouse brain.

21. Day 3: Label plastic molds for frozen block preparation with a permanent marker. It is necessary to make marks on the mold to indicate the orientation of the brain. Place a piece of clear tape over the labeling to prevent smearing. Place the molds on a flat surface, cover the bottom of the mold with the NEG50 or OCT freezing compound.
22. Prechill acetone in a Petri dish placed on dry ice pellets. Keep this Petri dish on top of dry ice inside a Styrofoam box.

**CRITICAL:** Tissues should be frozen as rapidly as possible to avoid ice crystals. The method we prefer uses dry ice in pellet form. Work in a fume hood. Put a minimal amount of acetone into a petri dish so the bottom is covered. Carefully add a couple small dry ice pellets to the dish with acetone, leaving enough room for the mold. When the pellets stop bubbling vigorously, the "slurry" is ready. To maintain the temperature, you might have to add more dry ice pellets. Once you have filled the mold and oriented the tissue, place it in the cold acetone to freeze it.

**CRITICAL:**This step is important for keeping the intact structure of the brain anatomy, including astrocytes morphology.

23. Remove the brain from the 30% sucrose solution. Use forceps to keep the brain in upright position. Hold a corner of the brain on a Kimwipe to release excess liquid and then place the brain on the empty dish on ice. Cut the bottom part of the brain to keep it level, if required.
24. Place the brain into molds filled with NEG50. Mix the brain gently with NEG50. Orient the brain as required for the most efficient sectioning. Cover the tissue with NEG50 and fill up the mold.
25. Freeze quickly by placing the mold on petri dish with acetone on dry ice. Make sure the acetone only goes to the bottom quarter of the mold. Allow it to stand on dry ice until the block is fully white and solid. Dry the bottom of the mold with a paper towel. Wrap the mold with aluminum foil or parafilm and label it. Store at −20°C for short storage (5 days or less) or −80°C for long term storage.

**Pause point:** Frozen blocks can be stored at −80°C for years. However, after freezing, the block should never be allowed to thaw. For transport, keep the blocks on dry ice.

### Cryosectioning, fixation, and permeabilization of the sections

**Timing: 3 days, 1–2 h each day; 2 h incubation**

This step describes how to section the frozen tissue and then fix and permeabilize the sections

26. Cryosectioning. Label the slides in order.
27. Set up the cryostat (Equipment Setup) and clean the stage. Use a new blade for sectioning.

**CRITICAL:** Keep frozen blocks inside the cryostat. Do not let the blocks thaw or even soften. However, blocks stored at −80°C must be allowed to warm to −20°C prior to sectioning.

28. Cut 10–15 μm thick frozen sections. Note: The suggested cryostat temperature is between −15°C and −23°C. The section will curl if the specimen is too cold. If it is too warm, it will stick to the knife.
29. Thaw-mount the sections onto Superfrost-coated positive charged histological slides.
30. Dry the slides for approximately 10 min on a slide warmer at 37°C. Slides containing cryostat sections can be stored at −20°C to −-80°C for up to 12 months.

**CRITICAL:** The specimen preparation technique greatly contributes to the histochemical properties of the tissue and hence the excellence of the subsequent histology. In this study, cryosectioning performed well in respect to the detection of intracellular peptide antigen like GFAP. The penetration of the IHC staining into the cryostat sections is key to good staining. A number of requirements regarding section thickness, staining intensity, and penetration must be carefully tested (Wirenfeldt et al., 2003). In the present study, GFAP-positive cells visualized better at 12 μm section.

***Note:*** The drying time is important to properly mount the tissue sample onto the slide. Insufficient drying may cause section detachment. To avoid RNA degradation, the drying time should not exceed 10 min.

31. Fixation and Permeabilization of the Sections: day 1. When staining cryostat sections stored in a freezer, thaw the slides at 20°C–22°C for 10–20 min. Procedures from here can be done in a coplin jar or slide holder.
32. Post-fix sections for 5 min with sterile 4% PFA in PBS.
33. Wash with sterile PBS, 2× 5 min each time.
34. Permeabilize sections for 2 min with 1 μg/mL RNase-free Proteinase K in PBS.
35. Wash with PBS, 1 × 5 min.
36. Re-fix sections for 5 min with 4% PFA in PBS.
37. Wash with PBS, 3× 5 min each time.
38. Incubate sections with 70% EtOH for 5 min.
39. Followed with 95% EtOH for 2 min.
40. Air dry in open humid chambers 5 min or until dry
41. Prepare hybridization probe in Hybridization Solution in dry dock at 80°C for 3 min.

***Note:*** RNA probe is generated based on standard protocol from Promega.

The majority of probes work between dilutions of 1:400–1:1,000 in hybridization buffer. Using two dilutions is helpful if probe produces high background. https://www.promega.com/products/cloning-and-dna-markers/molecular-biology-enzymes-and-reagents/t3-rna-polymerase/?catNum=P2083

42. Pipette 250–300 μL/slide of probe and place a parafilm coverslip over the sections. Place slides in humid chamber and incubate for 12–18 h at 65°C.
43. Day 2: Turn on water baths 37°C and 65°C
44. Remove coverslips from slides by immersing in 5× SSC. Use fine forceps if needed. Slides are now back in coplin jar.
45. In water bath at 65°C, wash sections with high-stringency wash for 30 min.
46. In water bath at 37°C, wash sections with RNase buffer for 3 × 10 min each time
47. To digest any single-stranded (unbound) RNA probe, incubate sections for 30 min at 37°C in RNase Buffer containing 20 μg/mL RNase A.
48. In water bath at 37°C, wash sections with RNase buffer for 15 min.
49. In water bath at 65°C, high-stringency wash sections for 2 × 20 min.
50. In water bath at 37°C, wash sections in 2× SSC for 15 min.
51. In water bath at 37°C, wash sections with 0.1× SSC for 15 min.
52. Wash the sections with PBT (PBS + 0.1% Tween 20) at 20°C–22°C for 15 min.
53. Cover slides for 120 min with 250–300 μL/slide blocking solution (10% NGS in PBT with 0.5 mg/mL Levamisol) in humid chamber.
54. Wash the slides with PBT at 20°C–22°C 4 × 5 min each time.
55. Cover the slides with 250–300 μL/slide anti-DIG antibody 1:2,500 with10% NGS in PBT in a humid chamber at 4°C for 12–18 h.
56. Day 3: Decant anti-DIG antibody and wash the slides in coplin jar with PBT 6 × over 5 h.
57. Wash sections with NTMT buffer 2 × 10 min each time.
58. Cover each sample slide with approximately 300 μL of BM-Purple exposure solution for 30 min to 16 h in a dark humid chamber until desired color has developed.
59. When color development is optimal (Figure 1), stop the color reaction by incubating the slides in PBS 2 × 5 min each time.

**Figure 1:**
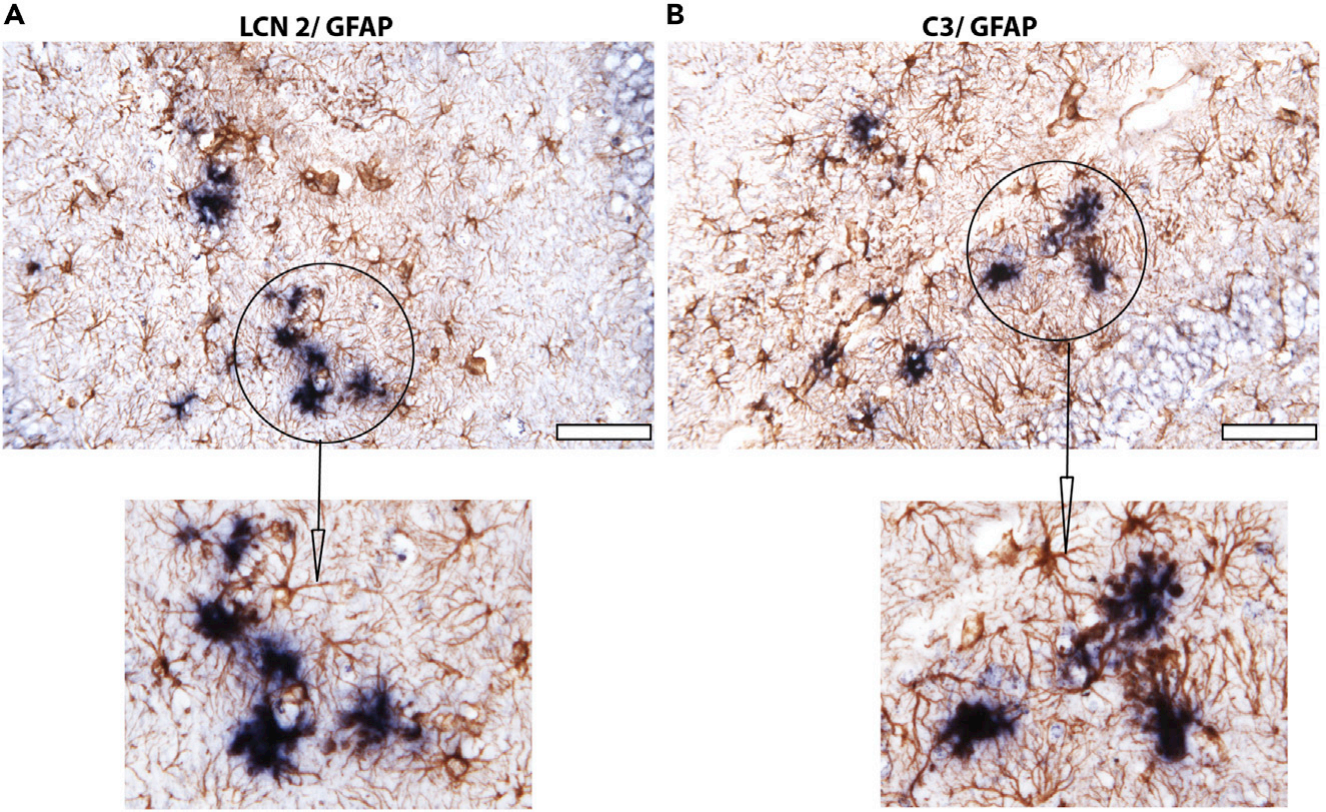
Example of combined IHC-*in situ* hybridization staining Combined IHC-*in situ* hybridization protocol shows expression of (A) LCN2 (lipocalin 2) and (B) C3 (complement C3) in astrocytes AMPK KO mouse astrocytes labeled with GFAP antibodies and hybridized with the RNA probes set for LCN2 and C3 shown in blue. Insets show magnifications of co-localization of LCN2 or C3 with GFAP. Scale bars, 50 μm.
60. Slides can be moved to IHC steps now

### Immunohistochemistry

**Timing: 2 days**

61. Wash sections in PBS for 5 min.
62. Wash with 0.3% H_2_O_2_ in PBS to inhibit endogenous peroxidase activity for 10 min at 20°C–22°C
63. Wash sections in PBS for 3 × 5 min each time.
64. Dry back of slide with tissue paper and add ∼325 μL anti-GFAP antibody (1:1,000) in blocking buffer and incubate for 12–18 h in humid chamber at 20°C–22°C.

**CRITICAL:** Antibody concentration needs to be optimized, as its critical for the success in GFAP staining. We systematically tested different GFAP antibodies and concentrations. In our hands, the staining result and the morphology of GFAP-positive cells visualized in this study are consistent with the original description of astrocytes.

65. Day 4: Wash sections in PBS for 3 × 5 min each time.
66. Add ∼325 μL biotinylated secondary antibody (1:500) in blocking buffer and incubate 2 h in humid chamber.
67. Wash sections in PBS for 3 × 5 min each time.
68. Incubated the sections in avidin and biotinylated horseradish peroxidase macromolecular complex (ABC reagent), 10 μL/mL each of reagents A and B and 10% Triton X-100 in 1 mL PBS for 1 h.
69. Wash sections in PBS for 3 × 5 min each time.
70. Add ∼300 μL DAB per slide. Carefully observe the color development through a microscope and stop the reaction when the desired color obtained. DAB prepared according to the manufacturer’s directions. https://vectorlabs.com/dab-peroxidase-hrp-substrate.html
71. Wash sections in PBS for 3 × 5 min each time.
72. Mount slides with appropriately sized coverslip and Vectashield antifade mounting medium

***Note:*** Any standardized protocol for imaging can be used. We image with a Leica light microscope DM2500 with a Lecia DMC6200 camera and perform image acquisition and processing in the Leica Acquisition Software (LASX) suite. All imaging data are analyzed by Image J.

### Quantification of the GFAP-positive astrocytes

Assessing astrocyte structure and morphology requires complementary approaches to assess varying levels of detail. Currently, there are multiple tools available to analyze and quantify the extremely complex astrocyte morphology (Parekh and Ascoli, 2013) (Kulkarni et al., 2015). The choice between options is normally based upon both the level of detail needed to answer the scientific question and the resources that are available. Reproducible visualization of astrocytes in brain is essential for quantitative studies of the cellular changes in CNS diseases. Lyck et al describe a detailed procedure to identify antibodies and protocols that could preserve and visualize astrocytes and neurons for quantitative stereological studies (Lyck et al., 2008). Another reliable protocol is to perform intracellular iontophoresis of astrocytes using fluorescent Lucifer yellow dye in lightly fixed brain tissue from adult mice (Moye et al., 2019). More recently, a method utilizes a genetically encoded membrane associated fluorescent marker of astrocytes, called lymphocyte-specific protein tyrosine kinase (Lck), which is tagged with green fluorescent protein (GFP)(Testen et al., 2020).

For the quantitative evaluation in this study, we identified astrocytes as typical gray matter bushy cells with numerous short and ramified processes; fibrous astrocytes were typical white matter star-shaped cells with long, thin, and relatively non-ramified processes. We only considered reactive astrocytes as LCN2 or C3 positive cells that co-stained with GFAP and exhibited a clear soma with minimum overlapping branches in the fields.

To calculate astrocyte density, we selected three consecutive visual fields under 20× objective. We counted the number of astrocytes using Image J Cell Counter plugin.

## Expected outcomes

Reactive gliosis consists of a rapid induction of gene expression after insult and earlier studies confirmed induced Lcn2 and C3 as markers of reactive astrocytes (Zamanian et al., 2012). Immunohistochemistry method for GFAP, and the use of RNA probes for LCN2 and C3 is attractive. However, for mouse brain this is problematic due to the highly nonspecific interaction and signal interference from the nonspecific binding, which generates a non-measurable signal.

A recent study published from our lab used a genetic loss-of-function approach producing a brain specific AMPKβ1/β2 deletion model to demonstrate that AMPK activity is required for cortical neurogenesis (Muraleedharan et al., 2020). Our study showed at 2 years of age, neuronal count, cortical thickness, and brain volume of Ampk KO mice were substantially reduced. At 24 months, the number of GFAP-positive astrocytes was greatly increased in the AMPK Knockout brain suggestive of reactive gliosis that occurs in response to chronic CNS pathology. The reactive nature of these astrocytes in AMPK-deficient brains was confirmed by combined IHC-*in situ* hybridization that showed increased expression of LCN2 (lipocalin 2) and C3 (complement C3) in GFAP-positive Astrocytes (Figure 1).

This labeling technique provides good signal upon hybridization under a standard microscope. The major benefit of combining these two techniques is that changes in gene expression in pathological situations or following physiological or pharmacological manipulations can be semi-quantified. Our method of identifying LCN2; GFAP double positive or C3; GFAP double positive reactive astrocytes can be integrated with immunohistochemical detection of neurons, oligodendrocytes, or microglia to investigate cell-cell interaction in the normal or pathological brain tissue sections.

## Quantification and statistical analysis

1. Draw boxes of same area over each region and count the selected region for GFAP-positive cells using the cell counter plugin in ImageJ. Every 12^th^ section is stained for GFAP and counted and quantified as the number of GFAP -positive cells.
2. For cell counts in cortical layers, count all cells in a 250-μm-wide columnar area from the white matter to the pial surface. Any cell where the morphology is not clearly visible should be excluded from analysis.
3. At least 5 sections in each group are analyzed. The same approach can be applied to GFAP/*Lcn2* or GFAP/*Comp.C3* combined *in situ*/IHC stained cells.

Statistics was performed using Prism software (version 4) (Muraleedharan et al., 2020). For all experiments, we used one-way ANOVAs to determine statistical significance, which was defined as p ≤ 0.05.

## Limitations

*In situ* hybridization provides transcriptomic information in the spatial cellular context. However, a few key limitations of the conventional ISH procedure become apparent. A disadvantage of applying *in situ* hybridization techniques is the difficulty in identifying targets that have RNA copies. One of the challenges in achieving high signal sensitivity for ISH is that the DAB reaction proceeds rapidly to completion and cannot be monitored and stopped when an optimal signal-to-noise ratio has been reached.

Another important concern of *in situ* hybridization is to establish whether RNA levels of a given gene can be used as proxies for the corresponding protein levels. Considering, several studies have showed that proteome and transcriptome abundances are not satisfactorily correlated to act as proxies for each other (Nagaraj et al., 2011; Payne, 2015). We have not checked the consistency between RNA level and protein level in our models, but there are several publications showed LCN2 and C3 mRNA levels correlate with protein levels in brain(Ip et al., 2011; Lian et al., 2016).

## Troubleshooting

### Problem 1

Poor morphology of the tissue, such as cracks or folds (step 22 in Step-by-step method details).

### Potential solution

This could be due to sample freezing too slowly or maintaining the wrong temperature during cryosectioning. Collect new samples and snap-freeze them.

### Problem 2

RNA signal indistinguishable from background (steps 43–59).

### Potential solution

Change reagent(s) that might be contaminated with RNase. Use filter tips when pipetting. It could be due to low hybridization temperature and/or low washing temperature. If your RNA length permits, try adding more probe. Verify and maintain correct hybridization temperature (65°C) and washing temperatures. For long probes or those with a high GC content, increase the temperature of the hybridization and wash steps to 68°C.

## Resource availability

### Lead contact

Further information and requests for resources and reagents should be directed to and will be fulfilled by the lead contact, Biplab DasGupta (biplab.dasgupta@cchmc.org ).

### Materials availability

This study did not generate new unique reagents.

### Data and code availability

This study did not generate/analyze [datasets/code].
